# Spontaneous coronary artery dissection in women in the generative period: clinical characteristics, treatment, and outcome—a systematic review and meta-analysis

**DOI:** 10.3389/fcvm.2024.1277604

**Published:** 2024-02-08

**Authors:** Svetlana Apostolović, Aleksandra Ignjatović, Dragana Stanojević, Danijela Djordjević Radojković, Miroslav Nikolić, Jelena Milošević, Tamara Filipović, Katarina Kostić, Ivana Miljković, Aleksandra Djoković, Gordana Krljanac, Zlatko Mehmedbegović, Ivan Ilić, Srdjan Aleksandrić, Valeria Paradies

**Affiliations:** ^1^ Clinic for Cardiology, University Clinical Center Nis, Nis, Serbia; ^2^ Medical Faculty, University of Nis, Nis, Serbia; ^3^ Department of Cardiology, Clinical Hospital Bežanijska Kosa, Belgrade, Serbia; ^4^ Medical Faculty, University of Belgrade, Belgrade, Serbia; ^5^ Clinic of Cardiology, University Clinical Center of Serbia, Belgrade, Serbia; ^6^ Department of Cardiology, Institute for Cardiovascular Diseases Dedinje, Belgrade, Serbia; ^7^ Department of Cardiology, Maasstad Hospital, Rotterdam, Netherlands

**Keywords:** spontaneous coronary artery dissection, pregnancy, female population in the generative period, treatment, outcome

## Abstract

**Introduction:**

Spontaneous coronary artery dissection (SCAD) is a non-traumatic and non-iatrogenic separation of the coronary arterial wall.

**Materials and methods:**

This systematic review and meta-analysis is reported following the PRISMA guidelines and is registered in the PROSPERO database. A literature search was focused on female patients in generative period (16–55 of age) with acute coronary syndrome (ACS) caused by SCAD, and comparison from that database NP-SCAD (spontaneous coronary artery dissection in non pregnant women) and P-SCAD (spontaneous coronary artery dissection in pregnant women).

**Results:**

14 studies with 2,145 females in the generative period with ACS caused by SCAD were analyzed. The median age was 41 years (33.4–52.3 years). The most common risk factor was previous smoking history in 24.9% cases. The most common clinical presentation of ACS was STEMI in 47.4%. Conservative treatment was reported in 41.1%. PCI was performed in 32.7%, and 3.8% of patients had CABG surgery. LAD was the most frequently affected (50.5%). The prevalence of composite clinical outcomes including mortality, non-fatal MI and recurrent SCAD was 3.3% (95% CI: 1.4–5.1), 37.7% (95% CI: 1.9–73.4) and 15.2% (95% CI: 9.1–21.3) of patients. P-SCAD compared to NP-SCAD patients more frequently had STEMI (OR = 3.16; 95% CI: 2.30–4.34; *I*^2^ = 64%); with the left main and LAD more frequently affected [(OR = 14.34; 95% CI: 7.71–26.67; *I*^2 ^= 54%) and (OR = 1.57; 95% CI: 1.06–2.32; *I*^2 ^= 23%)]; P-SCAD patients more frequently underwent CABG surgery (OR = 6.29; 95% CI: 4.08–9.70; *I*^2 ^= 0%). NP-SCAD compared to P-SCAD patients were more frequently treated conservatevly (OR = 0.61; 95% CI: 0.37–0.98; *I*^2 ^= 0%). In P-SCAD compared to NP-SCAD mortality rates (OR = 1.13; 95% CI: 0.06–21.16; *I*^2 ^= not applicable) and reccurence of coronary artery dissection (OR = 2.54; 95% CI: 0.97–6.61; *I*^2 ^= 0%) were not more prevalent.

**Conclusion:**

The results of this meta-analysis indicated that patients with P-SCAD more frequently had STEMI, and events more frequently involved left main and LAD compared to NP-SCAD patients. Women with NP-SCAD were significantly more often treated conservatively compared to P-SCAD patients. P-SCAD compared to NP-SCAD patients did not have significantly higher mortality rates or recurrent coronary dissection.

## 1 Introduction

Spontaneous coronary artery dissection (SCAD) is a non-traumatic and non-iatrogenic separation of the coronary arterial wall and an infrequent cause of acute myocardial infarction. It is more common in younger females than in other general population groups. Potential predisposing factors include fibromuscular dysplasia (FMD), partum and postpartum period, multi-parity (≥4 births), connective tissue disorders, systemic inflammatory conditions, mental stress and hormonal therapy. While uncommon, SCAD should be considered in any young patient, especially young women without a history of coronary heart disease or traditional risk factors, who presents with an acute myocardial infarction or cardiac arrest (1, 2).

Two potential mechanisms for spontaneous coronary artery dissection were described: the intimal tear hypothesis and the medial hemorrhage hypothesis. Once the SCAD happens, due to weakness of the arterial wall, dissection can further propagate anterograde and retrograde (3).

Although SCAD is most often observed in women's reproductive period, it is not yet clear whether there are differences in clinical presentation, treatment, and outcomes in pregnant women or soon after delivery, compared to other women in reproductive period. The high progesterone level during pregnancy is usually associated with SCAD because of its role in the fragility of the arterial media through the replacement of the elastic fiber and mucopolysaccharide substances and in the reduction of collagen synthesis (4, 5). Hemodynamic changes during pregnancy can also provoke SCAD. The increased cardiac output and circulatory volume during pregnancy can cause structural changes in the aorta, which can also expand to the coronary arteries (6). Some studies reported that hormonal changes with lactation may compound the effects of pregnancy (7). In patients with SCAD history, there is a risk of SCAD recurrence during pregnancy or postpartum (8).

Early diagnosis of SCAD is important because the management of SCAD differs from the atherosclerotic disease. Urgent coronary angiography is the first-line imaging for patients presenting with acute coronary syndrome (ACS). However, coronary angiography has significant limitations in diagnosing SCAD because it does not show the structure of the arterial wall. Optical coherent tomography (OCT) and intravascular ultrasound (IVUS) that image the arterial wall layers may provide further information and improve SCAD diagnosis. Still, it is not widely available and is associated with additional risks and costs (1).

The optimal management of SCAD is still unknown. All recommendations are based on expert opinions on treating individual and serial cases of SCAD. Nowadays, progress in the field of SCAD is being made by the National Registries of SCAD cases with detailed risk factors, diagnostic procedures, and treatment recommendations. This meta-analysis aims to provide a comprehensive contemporary update of SCAD assisting healthcare professionals in recognizing and managing these patients promptly and effectively. A special effort is put into detailed analysis and comparison of risk factors, coronary angiography findings, treatment, and prognosis between pregnant females with SCAD (including the three months after delivery—postpartum), labelled as P-SCAD and non-pregnant females with SCAD, labelled as NP-SCAD to facilitate early diagnostics during pregnancy or even before pregnancy in vulnerable women.

## 2 Material and methods

This systematic review and meta-analysis is reported following the Preferred Reporting Items for Systematic Reviews and Meta-Analyses (PRISMA) recommendations (9) and is registered in the PROSPERO database (CRD42023424806).

### 2.1 Inclusion criteria

This study included the SCAD female population in the generative period.

Inclusion criteria were: (1) females in the generative period (16–55 years) with acute myocardial infarction (AMI) caused by SCAD occurring during pregnancy or within three months post-partum; (2) the diagnosis of SCAD confirmed by coronary angiography (10), (4) for analysis we included observational studies, randomized controlled trials, quasi-randomized controlled trials, non-randomized controlled trials, prospective and retrospective cohort studies.

Studies were excluded if: (1) postmenopausal women patients were included; (2) a male population was included and not reported subgroup analysis by gender; (3) case reports and literature reviews; (4) studies that investigated only iatrogenic coronary artery dissections; and (5) individual case series included in literature reviews were excluded to avoid double counting of results, as were restatements of prior studies that contained duplicative results.

### 2.2 Study selection

Two reviewers independently conducted searches on all information sources. The comprehensive search and selection process was ensured by using Rayyan QCRI software (https://rayyan.qcri.org). The comprehensive search and selection process was ensured using Rayyan QCRI software. A third reviewer (SA) identified and removed duplicates and ensured independent review of titles and abstracts by blinding decisions.

**Figure 1:**
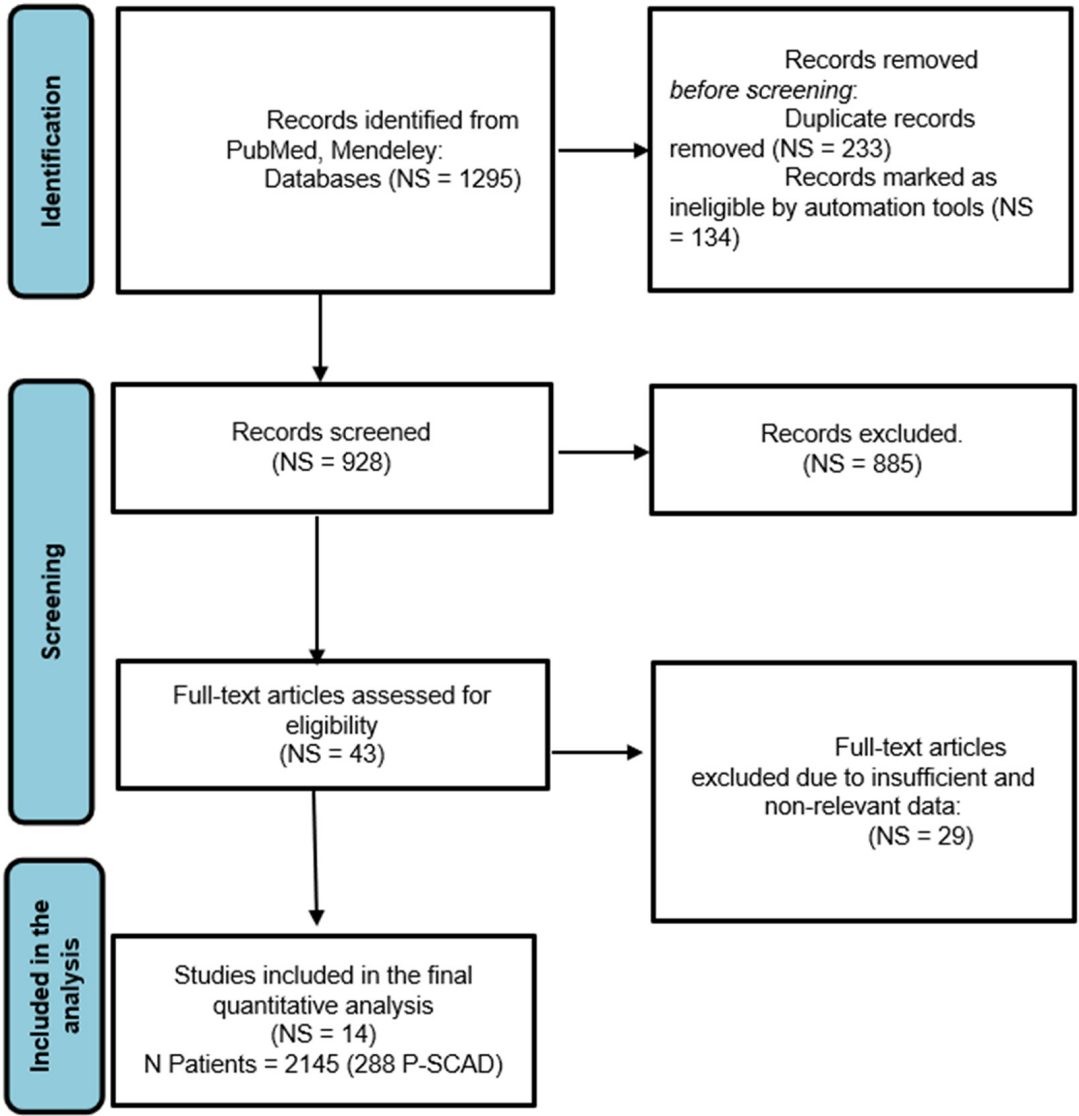
PRISMA flow chart of the systematic literature review and article identification process included in the meta-analysis. NS, number of analyzed studies with acute myocardial infarction and SCAD; *N*, number of total SCAD female patients; P-SCAD, pregnancy related spontaneous coronary artery disease.

In the first step of selection, the title and abstract were examined, and in the next step, when necessary, the full articles were obtained and read. Additional studies were identified through reference and citation tracking. Only articles in English were screened. Four reviewers independently screened the title, abstract and full text. Disagreement about including studies was resolved by discussion and consensus between the reviewers and collaborators (SA, AI). The study inclusion process is presented using the PRISMA flowchart (Figure 1).

### 2.3 Search strategy

We comprehensively searched electronic databases, including MEDLINE and Mendeley, limited to English-language publications. The initial search was performed on 04 April 2023 and repeated on 07 June 2023 to ensure up-to-date results. Relevant literature for this review was obtained by combining MeSH terms and keyword searches. These terms were further combined using “OR” or “AND” Boolean operators, and the use of $/* was employed where applicable. For more details on the search strategy, please refer to Appendix 1.

### 2.4 Data collection process

Data extraction was conducted from the included studies, covering the characteristics of the study population, study design, demographic and clinical characteristics of SCAD, risk factors, clinical presentation, treatment and management, outcomes, coronary territory, and obstetrical history. The extracted data were systematically organized into tables and compared. The study's primary outcomes focused on clinical presentations, treatment and management, coronary territory, and outcomes (deaths, recurrent SCAD) in SCAD females. Missing data were not input into the analysis.

A quality assessment was carried out using the Downs and Black tools. The Downs and Black score, ranging from 0 to 27, was categorized into three tiers: good (≥20), fair (15–19), and poor (≤14).

Subgroup analysis was performed to compare age, presence of STEMI, conservative treatment, CABG, and death and recurrent SCAD between pregnant SCAD and non-pregnant SCAD females. Although we intended to assess BMI and PCI, these outcomes were not reported in studies with P-SCAD. Unfortunately, sensitivity analysis could not be performed due to the limited number of included studies.

### 2.5 Statistical analysis

The outcomes were treated as a dichotomous variable: presence of risk factors (yes/no), clinical presentations (yes/no), treatment and management (conservative treatment (yes/no), CABG (yes/no), recurrent SCAD (yes/no), with respective 95% confidence interval (95% CI). Statistical heterogeneity was assessed with the *I*^2^ statistic, and significance was assumed when the *I*^2^ was greater than 50%. The *I*^2^ statistic illustrates the percentage of the variability in effect estimates resulting from heterogeneity rather than sampling error. In the first part, we performed a proportional meta-analysis of the prevalence of risk factors, baseline characteristics, clinical presentation of ACS, treatment, SCAD coronary territory and outcomes using Der Simonian–Laird binary random or Peto fixed-effect meta-analysis in Open Meta. Results from the proportional meta-analysis were tabulated and graphically displayed in Table 7. Secondly, we compared risk factors, clinical presentation of ACS, treatment, coronary territory and outcomes between pregnant SCAD and non-pregnant SCAD using Peto fixed-effect meta-analysis in Review Manager Version 5.4.1. A *p*-value <0.05 was considered significant. Subgroup analyses were graphically presented by forest plot. Publication bias was not estimated following the recommendations for proportional meta-analysis (11).

## 3 Results

Figure 1 shows the PRISMA flow chart, from search and identification studies to inclusion in the meta-analysis. After removing duplicates, the abstracts of 928 articles were screened. In the screening process, 885 articles were excluded. The full text of the remaining 43 articles was assessed for eligibility. Of these, 29 studies containing male and female patients with SCAD were excluded because risk factors, treatment, and outcome data were not differentiated between men and women. Hence, fourteen (7, 8, 12–17, 18, 19–30) studies were included in the quantitative synthesis, with a population of 2,145 females in the generative period with ACS caused by SCAD (257 pregnancy—associated with SCAD—12.0% of patients) (Table 1). The general timeline of these studies ranged from 2011 to 2021. The median age of the female (in the generative period) SCAD population was approximately 41 years (33–52 years).

**Table 1:** Design of included studies, number of pregnancy-related spontaneous coronary artery dissection (P-SCAD) and nonpregnancy-related SCAD of women in the generative period (NP-SCAD) and quality of the studies.

Authors	Year	Country	Study design	Study population	No of generative period female SCAD patients	P-SCAD	Quality assessment
Nakashima et al. (7)	2016	Netherlands	Cohort study	20,195	45	5	15
Daoulah et al. (40)	2021	United States	Observational study	83	42	12	16
Tweet et al. (8)	2017	United States	Mayo SCAD registry	323	323	88	12
Ito et al. (12)	2011	United States	Case series, retrospective study	23	23	7	13
Vautrin et al. (61)	2020	United States	Cohort study	144	51	0	13
Fahmy et al. (17)	2016	United States	Cohort study	288	263	0	12
Tweet et al. (15)	2014	United States	Retrospective study	189	174	26	16
Cauldwell et al. (19)	2020	England	Multicenter retrospective study	79	2	2	13
Havakuk et al. (18)	2017	United States	Case series, retrospective study	120	120	84	11
Chen et al. (30)	2021	United States	Case series, retrospective study	307	307	0	13
Faden et al. (16)	2016	England	Cohort	79	79	0	15
Tweet et al. (14)	2012	United States	Retrospective study	87	71	13	13
Tweet et al. (62)	2020	United States	Cohort study	636	636	18	18
Toggweiler et al. (13)	2012	Switzerland	Cohort study	12	9	2	9

P-SCAD, pregnant spontaneous coronary artery dissection, quality assessment was carried out using the Downs and Black tools.

Analyzed characteristics of included studies: baseline clinical characteristics, clinical presentations ACS, risk factors, treatment, SCAD coronary territory, and outcomes are presented in Tables 1–6. The quality of the studies was generally poor and fair, ranging from 9 to 18 (median 13, average 13.5). Of the 14 studies examined, five were fair quality, scoring 15–18, and nine were poor quality, scoring 9–13 on the modified Downs and Black scale. Meta-analysis was conducted on baseline characteristics, clinical presentation of ACS, treatment, SCAD coronary territory, and in-hospital outcomes (Table 7). The most common risk factors were smoking history 24.9% (95% CI: 13–36.8) and hypertension 22.1% (95% CI: 11.3–32.9). There is not enough data to make a difference in the prevalence of smoking history and hypertension between pregnant and non-pregnant patients. There are no data on whether a new-onset increment of blood pressure was the reason for the occurrence of SCAD or whether the patient was treated for hypertension before. Occurrences of dyslipidemia and diabetes mellitus in analyzed female patients in the generative period with SCAD were 19.4% (95% CI: 9–29.7) and 3.3% (95% CI: 1.5–5.1). The most common clinical presentations of acute coronary syndrome were STEMI in 47.4% (95% CI: 28.5–66.2) and NSTEMI in 39.8% (95% CI: 15.2–64.4) of cases. Conservative treatment was used in 41.1% of patients (95% CI: 23.2–59.1). The percutaneous coronary intervention (PCI) was performed in 32.7% (95% CI: 19.9–45.4), and coronary artery bypass grafting (CABG) was done in 3.8% (95% CI: 0.2–5.7) of patients. The most affected artery was LAD in 50.5% (95% CI: 21.9–79.1). Multivessel SCAD was diagnosed in 15.5% (95% CI: 8.6–22.3) of patients. In the analysis of the clinical outcomes, including mortality, non-fatal MI and recurrent SCAD, the prevalence of mortality was 3.3% (95% CI: 1.4–5.1), while non-fatal MI and recurrent SCAD had 37.7% (95% CI: 1.9–73.4) and 15.2% (95% CI: 9.1–21.3) of included patients (Table 7).

**Table 2:** Summary of age, risk factors and comorbidities of reviewed patients with spontaneous coronary artery dissection (SCAD) in the included studies.

Authors	Nakashima et al. (7)	Daoulah et al. (40)	Tweet et al. (8)	Ito et al. (12)	Vautrin et al. (61)	Fahmy et al. (17)	Tweet et al. (15)	Cauldwell et al. (19)	Havakuk et al. (18)	Chen et al. (30)	Faden et al. (16)	Tweet et al. (14)	Tweet et al. (62)	Toggweiler et al. (13)
Age ± SD, years	41 ± 7	39 ± 13	41 ± 38	45 ± 11	39 ± 5	52 ± 9	44 ± 9	30 ± 6	34 ± 4	44 ± 8	33 ± 5	42 ± 9	38 ± 4	47 ± 9
BMI ± SD, kg/m^2^	22.5 ± 4.6	29.0 ± 5.4	25.5 ± 5.0	NA	NA	NA	NA	NA	NA	NA	NA	26.2 ± 5.7	25.0 ± 6.0	NA
Hypertension, *n*	10	7	86	13	9	87	NA	NA	5/95^a^	99	NA	13	6	NA
Smoking, *n*	18	6	86	7	36	32	NA	NA	12/96^a^	NA	NA	NA	4	NA
Dyslipidemia, *n*	6	12	113	5	16	53	NA	NA	9/94^a^	81	NA	7	4	NA
Diabetes mellitus, *n*	NA	6	3	1	4	8	NA	NA	4/95^a^	24	NA	2	1	NA
FMD, *n*	NA	NA	161	NA	NA	191	NA	NA	NA	NA	NA	10	6	NA
Emotional stress, *n*	NA	26	43	4	NA	144	NA	NA	NA	NA	NA	NA	9	NA
Migraines, *n*	NA	17	47	NA	NA	NA	NA	NA	NA	NA	NA	NA	9	NA
Thyreoidism, *n*	NA	NA	51	NA	NA	NA	NA	NA	NA	NA	NA	NA	2	NA
IVF, *n*	NA	NA	15	NA	NA	NA	NA	NA	NA	6	NA	1	3	NA
Hormonal therapy, *n*	1	5	59	NA	NA	NA	NA	NA	7/38 (?)	18	NA	11	NA	NA
Contraception, *n*	0	NA	NA	2	NA	NA	NA	NA	NA	NA	NA	5	NA	NA
Eclampsia, *n*	NA	NA	1	NA	NA	NA	NA	NA	8/65 (?)	NA	NA	NA	NA	NA

NA, not available—unavailability of data for the examined group of female patients in the generative period; BMI, body mass index; FMD, fibromuscular dysplasia; IVF, *in vitro* fertilization.

^a^ Data were not provided for all the patients.

**Table 3:** Clinical presentations of ACS overall in pregnancy-related spontaneous coronary artery dissection (P-SCAD) and nonpregnancy-related SCAD of women in the generative period (NP-SCAD) in the reviewed studies (summary of acute coronary syndrome SCAD).

Authors	Unstable angina	Cardiac arrest	STEMI	NSTEMI
Nakashima et al. (7)	NA	1/45	39/45	NA
Daoulah et al. (40)	24/42	NA	24/42	18/42
Tweet et al. (8)	8/323	33/323	128/323	186/323
Ito et al. (12)	11/23	1/23	11/23	11/23
Vautrin et al. (61)	NA	1/51	51/51	0/51
Fahmy et al. (17)	NA	NA	73/263	190/263
Tweet et al. (15)	NA	NA	NA	NA
Cauldwell et al. (19)	NA	NA	NA	NA
Havakuk et al. (18)	28/120	NA	87/120	28/120
Chen et al. (30)	NA	NA	89/370	NA
Faden et al. (16)	NA	9/79	42/79	24/79
Tweet et al. (14)	5/71	NA	32/71	34/71
Tweet et al. (62)	1/636	NA	8/636	14/636
Toggweiler et al. (13)	NA	NA	6/9	3/9

NA, not available, unavailability of data for the examined group of female patients in the generative period; ACS, acute coronary syndrome; SCAD, spontaneous coronary artery dissection; P-SCAD, pregnancy-related SCAD; NP-SCAD, non-pregnancy related SCAD; STEMI, ST-elevation myocardial infarction; NSTEMI, non-ST-elevation myocardial infarction.

**Table 4:** Aggregate initial treatment of pregnancy-related spontaneous coronary artery dissection (P-SCAD) and nonpregnancy-related SCAD of women in the generative period (NP-SCAD) cases in the reviewed studies (summary of treatment of reviewed studies).

Authors	Nakashima et al. (7)	Daoulah et al. (40)	Tweet et al. (8)	Ito et al. (12)	Vautrin et al. (61)	Fahmy et al. (17)	Tweet et al. (15)	Cauldwell et al. (19)	Havakuk et al. (18)	Chen et al. (30)	Faden et al. (16)	Tweet et al. (14)	Tweet et al. (62)	Toggweiler et al. (13)
Number of female SCAD patients	45	42	323	23	51	263	174	2	120	307	79	71	636	9
Conservative treatment	27	21	NA	14	0	225	87	1	54	89	8	37	13	NA
Stent	NA	21	NA	4	22	38	82	1	37	68	31	43	9	NA
CABG	NA	2	NA	4	2	0	NA	NA	27	3	26	7	2	NA
BB	NA	38	NA	NA	40	NA	NA	2	NA	NA	NA	NA	NA	NA
ACEI	NA	23	NA	NA	14	NA	NA	NA	NA	NA	NA	NA	NA	NA
Ca ant	NA	3	NA	NA	NA	NA	NA	NA	NA	NA	NA	NA	NA	NA
Aminoacyl acid therapy	NA	4	NA	NA	50	NA	NA	2	NA	NA	NA	NA	NA	NA
Purinergic receptor P2Y, G-protein coupled, 12 protein therapy	NA	42	NA	NA	47	NA	NA	2	NA	NA	NA	NA	NA	NA
Anticoagulant therapy	NA	NA	NA	NA	38	NA	NA	NA	NA	NA	NA	NA	NA	NA

NA, not available—unavailability of data for the examined group of female patients in the generative period; SCAD, spontaneous coronary artery dissection; CABG, coronary artery bypass graft surgery; BB, beta blockers; ACEI, angiotensin-converting enzyme inhibitors; Ca ant, Calcium antagonist.

**Table 5:** Vessel involvement overall in pregnancy-related spontaneous coronary artery dissection (P-SCAD) and nonpregnancy-related SCAD of women in the generative period (NP-SCAD) in the reviewed studies (summary of SCAD coronary territory were involvement of reviewed studies).

Authors	Nakashima et al. (7)	Daoulah et al. (40)	Tweet et al. (8)	Ito et al. (12)	Vautrin et al. (61)	Fahmy et al. (17)	Tweet et al. (15)	Cauldwell et al. (19)	Havakuk et al. (18)	Chen et al. (30)	Faden et al. (16)	Tweet et al. (14)	Tweet et al. (62)	Toggweiler et al. (13)
Number of female SCAD patients	45	42	323	23	51	263	174	2	120	307	79	71	636	9
LAD, *n*	NA	17	199	16	28	NA	NA	1	NA	168	NA	51	13	NA
LCX, *n*	4	5	47	6	4	NA	25	NA	28	108	NA	13	4	NA
LM, *n*	0	8	26	1	0	NA	4	NA	43	7	NA	8	2	NA
RCA, *n*	15	9	46	5	12	NA	25	NA	18	69	NA	21	4	NA
Multivessel, *n*	7	2	57	6	7	NA	15	NA	48	34	NA	18	6	NA

NA, not available—unavailability of data for the examined group of female patients in the generative period. SCAD, spontaneous coronary artery dissection; LAD, left anterior descending artery; LCX, circumflex artery; LM, left main coronary artery; RCA, right coronary artery.

**Table 6:** Findings of early outcomes of patients with spontaneous coronary artery dissection—overall in pregnancy-related spontaneous coronary artery dissection (P-SCAD) and nonpregnancy-related SCAD of women in the generative period (NP-SCAD) in the reviewed studies (summary of outcomes of reviewed studies (intrahospital/short outcome).

Authors	Nakashima et al. (7)	Daoulah et al. (40)	Tweet et al. (8)	Ito et al. (12)	Vautrin et al. (61)	Fahmy et al. (17)	Tweet et al. (15)	Cauldwell et al. (19)	Havakuk et al. (18)	Chen et al. (30)	Faden et al. (16)	Tweet et al. (14)	Tweet et al. (62)	Toggweiler et al. (13)
Number of female SCAD patients	45	42	323	23	51	263	174	2	120	307	79	71	636	9
Composite endpoint, *n*	17	NA	NA	NA	NA	NA	NA	6	NA	85	NA	NA	NA	NA
Reduced EF, *n*	NA	NA	16	NA	NA	NA	NA	NA	NA	NA	NA	NA	NA	NA
Death, n	1	NA	0	0	1	51	NA	3	5	1	3	NA	NA	NA
Non-fatal MI, *n*	16	NA	NA	2	8	NA	NA	1	NA	NA	66	NA	NA	NA
Recurrent SCAD, *n*	12	NA	33	1	NA	NA	NA	2	NA	36	NA	15	NA	2
Progression of residual SCAD, *n*	12	NA	NA	1	NA	NA	NA	NA	NA	44	NA	NA	NA	NA
Urgent revascularization, *n*	0	NA	20	NA	NA	NA	NA	NA	NA	NA	NA	NA	NA	NA

NA, not available—unavailability of data for the examined group of female patients in the generative period; SCAD, spontaneous coronary artery dissection; EF, ejection fraction; MI, myocardial infraction.

**Table 7:** Meta-analysis of the prevalence of risk factors, treatment, involvement in coronary territory and early outcomes—pooled studies.

	No. of studies	Total cases	Prevalence [95% CI]		*I*^2^ (%)	*p*-value
Baseline characteristics						
Hypertension	10	335	22.1 [11.3–32.9]	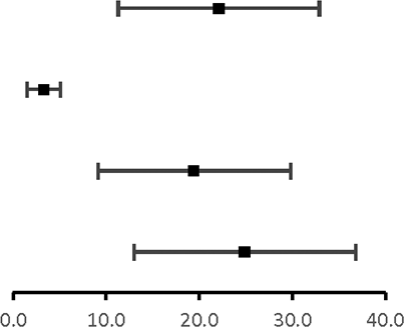	97.8	<0.001
Diabetes mellitus	9	53	3.3 [1.5–5.1]		83.8	<0.001
Dyslipidemia	10	306	19.4 [9–29.7]		97.6	<0.001
Smoking	8	201	24.9 [13–36.8]		97.7	<0.001
					
Clinical presentation						
Unstable angina	6	77	14.6 [8.6–20.7]	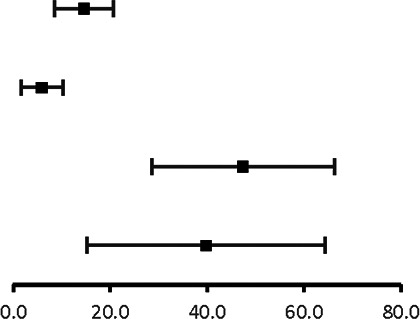	95.9	<0.001
Cardiac arrest	5	45	5.9 [1.6–10.2]		74.9	0.003
STEMI	11	539	47.4 [28.5–66.2]		99.1	<0.001
NSTEMI	9	508	39.8 [15.2–64.4]		99.3	<0.001
					
Treatment and management						
Conservative	12	579	41.1 [23.2–59.1]	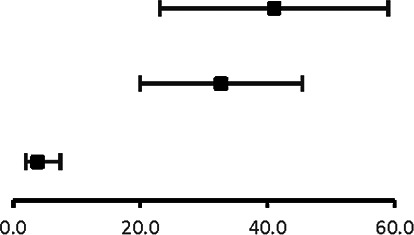	99.4	<0.001
Stent	11	356	32.7 [19.9–45.4]		97.9	<0.001
CABG	9	73	3.8 [0.2–5.7]		90.9	<0.001
					
Coronary territory						
LAD involvement	8	493	50.5 [21.9–79.1]	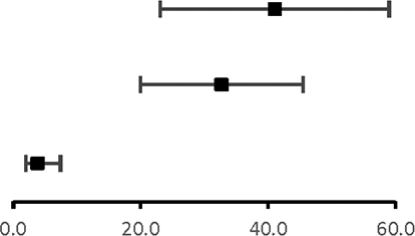	99.3	<0.001
Lcx involvement	10	244	15.8 [7.2–24.4]		96.9	<0.001
Left main involvement	10	99	5.9 [3.1–8.8]		92.1	<0.001
RCA involvement	10	224	18.9 [10.7–27.2]		96.3	<0.001
Multivessel	10	200	15.5 [8.6–22.3]		95.6	<0.001
					
Outcomes						
Death	8	62	3.3 [1.4–5.1]	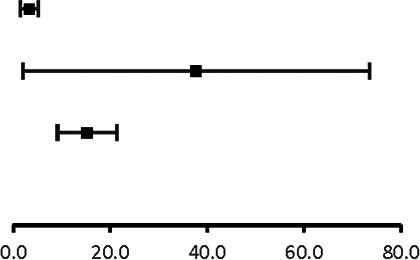	90.1	<0.001
Non-fatal IM	5	93	37.7 [1.9–73.4]		97.5	<0.001
Recurrent SCAD	7	101	15.2 [9.1–21.3]		75.3	<0.001

SCAD, spontaneous coronary artery dissection; CABG, coronary artery bypass graft surgery; LAD, left anterior descending artery; LCX, circumflex artery; LM, left main coronary artery; RCA, right coronary artery; STEMI, ST-elevation myocardial infarction; NSTEMI, non-ST-elevation myocardial infarction; IM, myocardial infarction

**Figure 2:**
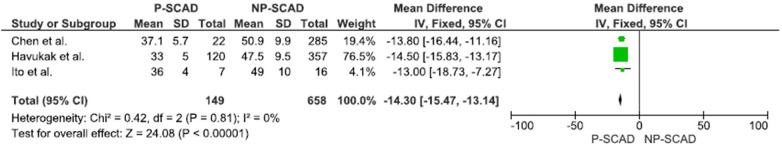
Forest plot age difference in pregnant and non-pregnant SCAD females.

**Figure 3:**
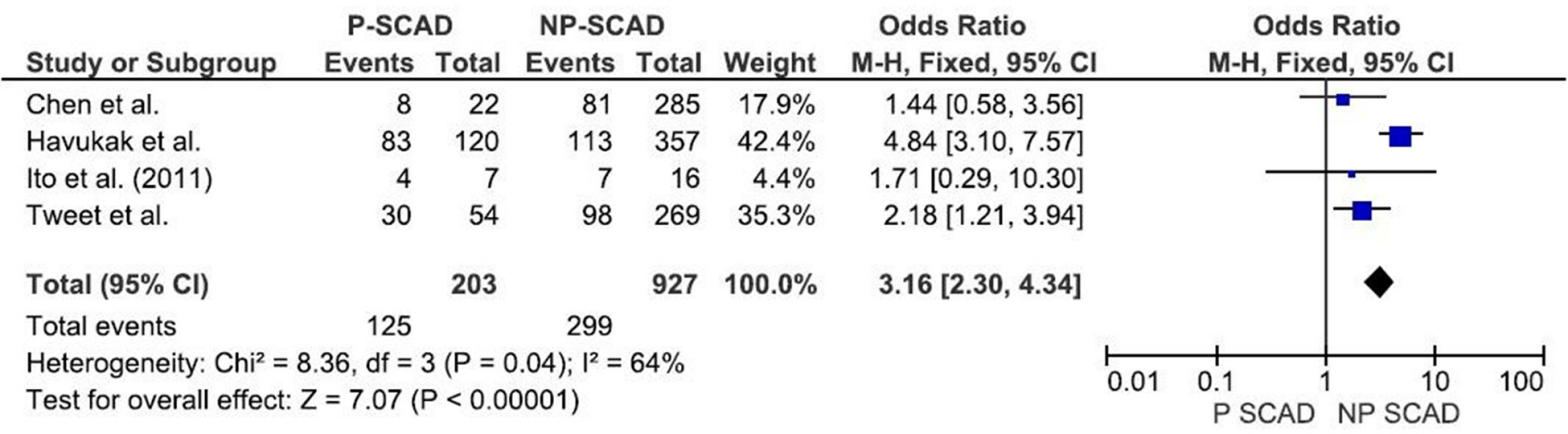
Forest plot for STEMI in pregnant and non-pregnant SCAD females.

**Figure 4:**
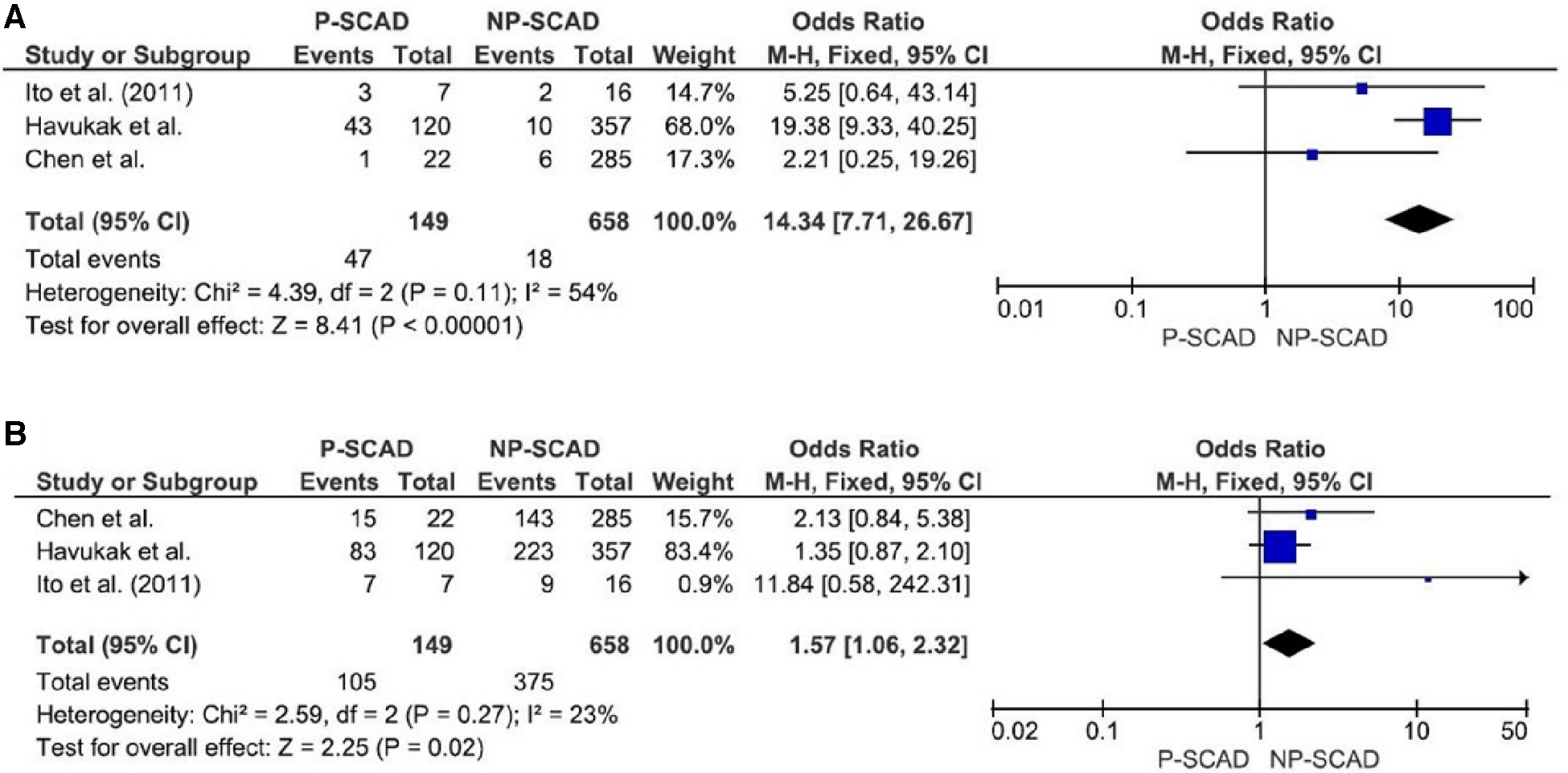
Forest plot for LM (**A**) and LAD (**B**) in pregnant and non-pregnant SCAD females.

**Figure 5:**
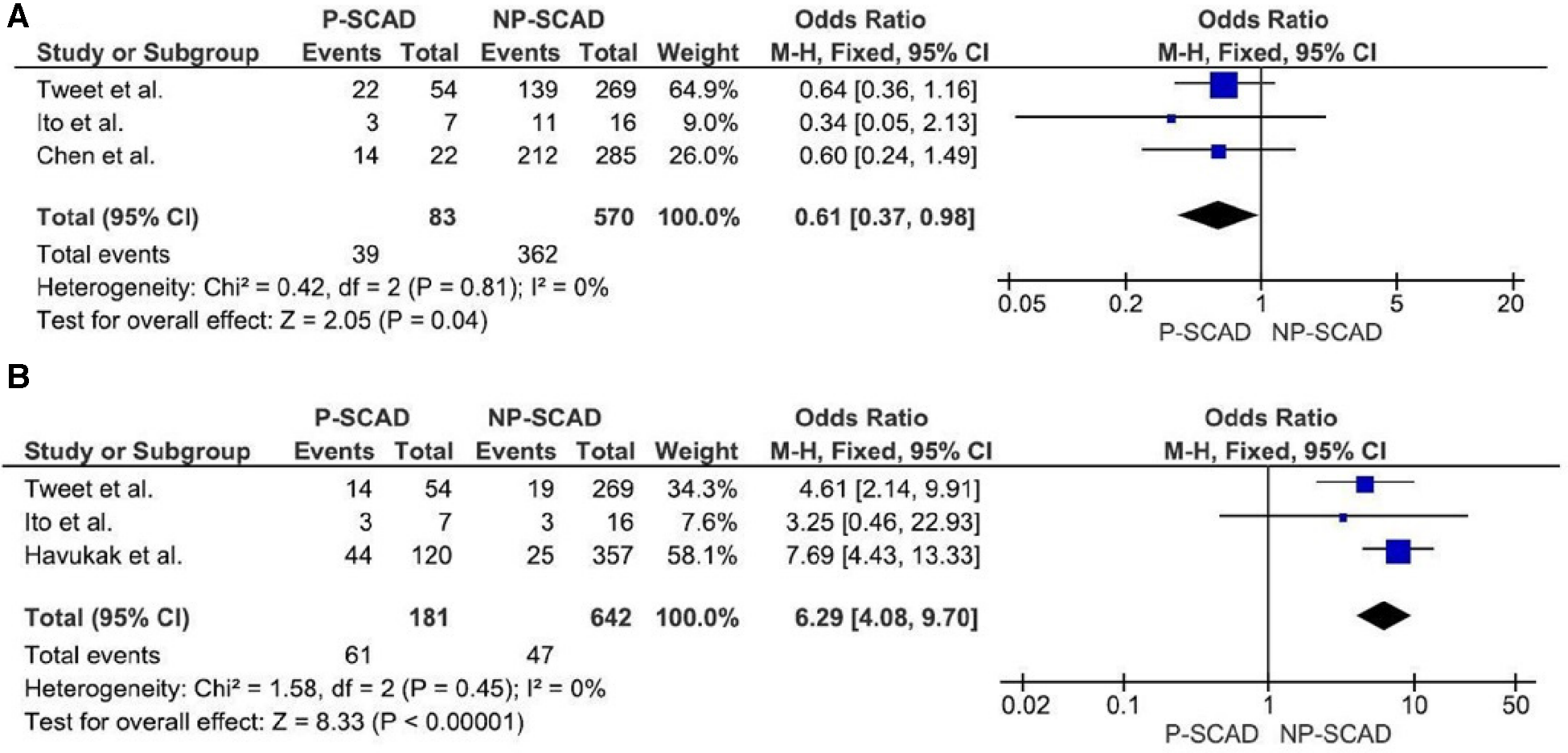
Forest plot for conservative management (**A**) and CABG (**B**) in pregnant and non-pregnant SCAD females.

**Figure 6:**
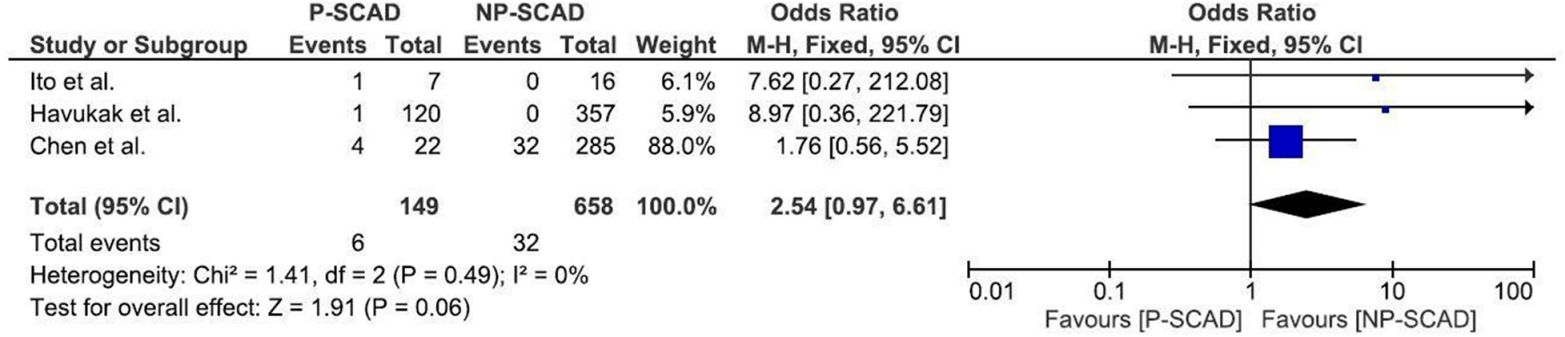
Forest plot for recurrent SCAD in pregnant and non-pregnant SCAD females.

**Figure 7:**
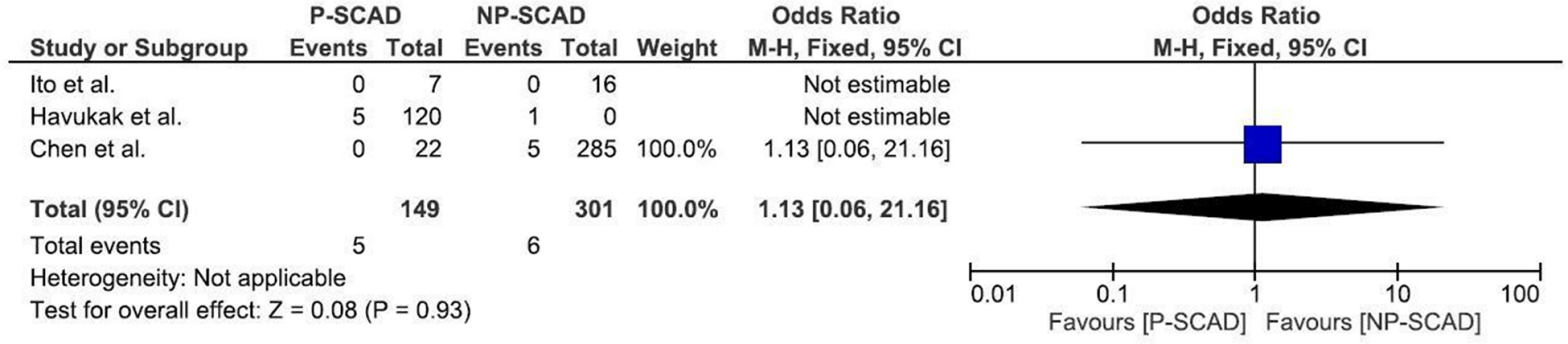
Forest plot for mortality in pregnant and non-pregnant SCAD females.

The analysis of pooled data showed a significant difference in age between P-SCAD and NP-SCAD patients (mean difference was 14.3 years, *p* < 0.001, *I*^2 ^= 0%), (Figure 2). The prevalence of STEMI was evaluated in 4 out of 14 studies. The meta-analysis result indicated that the prevalence of STEMI was significantly more frequent in patients with P-SCAD compared to those with NP-SCAD (OR = 3.16; 95% CI: 2.30–4.34; *I*^2 ^= 64%) (Figure 3). The prevalence of affected left main was evaluated in 3 out of 14 studies. The meta-analysis result indicated that the prevalence of left main involvement was significantly more frequent in P-SCAD compared to women with NP-SCAD (OR = 14.34; 95% CI: 7.71–26.67; *I*^2 ^= 54%) (Figure 4). The prevalence of LAD involvement was evaluated in 3 out of 14 studies. The meta-analysis result indicated that the LAD was more frequently affected in women with P-SCAD compared to those with NP-SCAD (OR = 1.57; 95% CI: 1.06–2.32; *I*^2 ^= 23%) (Figure 4). The prevalence of conservative management was evaluated in 3 of 14 studies. The meta-analysis result indicated that the prevalence of conservative management was significantly higher in NP-SCAD vs. P-SCAD patients (OR = 0.61; 95% CI: 0.37–0.98; *I*^2 ^= 0%) (Figure 5). The prevalence of CABG was evaluated in 3 of 14 studies. The meta-analysis result indicated that the higher prevalence of CABG was reported in P-SCAD patients compared to another group (OR = 6.29; 95% CI: 4.08–9.70; *I*^2 ^= 0%) (Figure 5). The prevalence of recurrent SCAD was evaluated in 3 of 14 studies. The meta-analysis result indicated that the prevalence of recurrent SCAD was not significantly higher in patients with P-SCAD compared to NP-SCAD cases (OR = 2.54; 95% CI: 0.97–6.61; *I*^2 ^= 0%) (Figure 6). Mortality was evaluated in 3 of 14 studies. The meta-analysis result indicated that the mortality was not significantly higher in women with P-SCAD vs. NP-SCAD cases (OR = 1.13; 95% CI: 0.06–21.16; *I*^2 ^= not applicable) (Figure 7).

## 4 Discussion

SCAD is an increasingly recognized presentation of AMI, especially in young women (20). A particularly vulnerable population of patients is represented by pregnant women and the period after childbirth, where the onset of myocardial infarction caused by SCAD poses a danger for both the mother and the child, with great uncertainty if another pregnancy is planned. Mortality from infarction caused by SCAD is not negligible, especially since no data from randomized studies would show us guidelines for treating such patients (21).

What do we know about SCAD in females in the generative period, especially the risk factors, the natural course of the disease and therapeutic options?

This meta-analysis analyzed 2,145 women with ACS SCAD in the reproductive period; from that number, 257 (12%) had P-SCAD. Most included studies reported overall risk factors in P-SCAD and or overall, for male and female patients with diagnosed SCAD. The limited available information precludes reaching definite conclusions regarding the risk factors for P-SCAD vs. NP-SCAD in the generative period. Only the analysis of the age of females in the generative period, clinical presentation of ACS, SCAD coronary territory, treatment and SCAD recurrence was sufficiently powered to detect differences between P-SCAD vs. NP-SCAD.

### 4.1 Risk factors and associated pathologies

Statistical data processing showed that smoking history is the most common risk factor 24.9% (95% CI: 13–36.8). There is insufficient data to differentiate the prevalence of smoking history between pregnant and non-pregnant patients, although some studies reported that female smokers have a 2-fold increased risk of myocardial infarction (22, 23). Female smokers taking oral contraceptives are reported to have a 7–100 fold increased risk of myocardial infarction (22). The association between smoking can be explained through increased oxidative stress and sympathetic activity, which may predispose patients to an increased risk of acute coronary syndrome (22–24).

We found that the second most common risk factor is hypertension 22.1% (95% CI: 11.3–32.9). There is insufficient data to differentiate the prevalence of hypertension between pregnant and non-pregnant patients. There is no information on whether the sudden rise in blood pressure was the reason for the appearance of SCAD or whether the patient had a previous history of treated hypertension (25).

The pooled data of prevalence of dyslipidemia and diabetes mellitus among analyzed female patients in the generative period were 19.4% (95% CI: 9–29.7) and 3.3% (95% CI: 1.5–5.1). Data that support the hypothesis about the association between dyslipidemia and diabetes mellitus and their role in causing SCAD is that adipose tissue is also an endocrine organ that produces hormones, peptides and nonpeptides that affect cardiovascular homeostasis. Adipose tissue is a significant source of estrogens, angiotensinogen and markers of chronic inflammation that can trigger acute coronary syndrome: tumor necrosis factor alpha, interleukin-6 and plasminogen activator inhibitor-1 (26).

SCAD is often associated with “few or no traditional cardiovascular risk factors” (14). SCAD patients have a lower prevalence of traditional cardiovascular risk factors than the national, age-matched average. It is known that some risk factors such as hypertension are similar with age matched national prevalence. Therefore, SCAD should be considered in the differential diagnosis of young men and women who present with ACS even in the presence of traditional risk factors (27). Patients with ACS caused by SCAD found to have high prevalence of hypothyroidism that those with atherosclerotic ACS (28). Freire et al. showed that hypothyroidism was more associated with diffuse and distal coronary lesions with SCAD, which were mostly managed conservatively (29). Also hypothyroidism has been associated with a higher frequency of iatrogenic coronary artery dissection during angioplasty (28). Although Faden et al. did not connect hypothyroidism with increased risk for SCAD (16).

The incidence of traditional risk factors for coronary artery disease (CAD) is lower or similar in pregnant patients with SCAD compared to those with atherosclerotic myocardial infarction at similar age (8, 12, 18, 23, 30). Zeven et al. found that patients at a third trimester have a highest risk for SCAD. In other studies the highest incidence of SCAD was reported in so called peripartum (which includes delivery and 1 week after delivery) and postpartum period up to 30 days after giving birth (2). The most frequent contributing factors for P-SCAD include genetics, hormonal influences, systemic inflammatory diseases, inherited or acquired arteriopathies, and environmental factors. Special attention is given to high levels of estrogen in P-SCAD which influences the arterial wall structure. Estrogen leads to increased activity of metalloproteases which can lead to weakening od arterial wall and its dissection. In accordance with the previous, the hormonal exposure during *in vitro* fertilization (IVF) is associated with an increased risk for vascular dissection in treated women during and after the IVF process (31, 32). Further, hormonal therapy was found to be a potential cause of SCAD in non-pregnant women (7, 18). We did not find publications where direct and significant correlation between the use of oral contraceptives and SCAD was determined.

### 4.2 Precipitating stressors

Some studies mark emotional stress as a trigger for SCAD because it correlates with catecholamines (7, 16, 33–37). It is believed that catecholamines may cause structural changes in the arterial wall leading to intimal rupture or disruption of the vasa vasorum, possibly through increased myocardial contractility or vasospasm (7). Some studies emphasize the association between migraine and arterial dissection, which is explained by the hypothesis of extracellular matrix defect (16, 38). Patients with migraine could be predisposed to vascular injury and endothelial dysfunction possibly due to genetic factors or hormones (39). The sample size and available data of precipitating stressors between the P-SCAD and NP-SCAD groups were too small to confirm the statistical contribution of all precipitating stressors to diagnosed SCAD.

### 4.3 Coronary territory

The main mechanism of myocardial injury in SCAD is ischemia induced by coronary artery narrowing of varying degrees due to intramural hematoma formation after intimal disruption (40). The main difference in the pathophysiology of different types of SCAD is the precipitating factors and causes that lead to arterial wall weakening (40). The meta-analysis result indicated that the prevalence of left main (LM) was significantly associated with P-SCAD (OR = 14.34; 95% CI: 7.71–26.67; *I*^2 ^= 54%). These findings are consistent with previous reports of LM involvement, including preliminary results of the Dissection of Coronary Arteries: Veneto and Emilia Registry (DISCOVERY) study (41).

The meta-analysis result indicated that the prevalence of LAD involvement is significantly more prevalent in P-SCAD group (OR = 1.57; 95% CI: 1.06–2.32; *I*^2 ^= 23%). Compared with non-pregnant women with SCAD, P-SCAD is associated with more extensive involvement of the coronary arteries manifested by a significantly higher rate of LM and multivessel dissections (12, 18). SCAD in postpartum involved more proximal coronary segments and LAD, which likely led to higher peak troponin I level, lower left ventricular ejection fraction, and more frequent congestive heart failure on presentation (12). In addition, there is a markedly higher incidence of STEMI and involvement of the left ventricle (LV) anterior wall, and as a result, a marked decrease in LV ejection fraction compared with non-pregnant patients. There is an increased incidence of cardiogenic shock, life-threatening arrhythmias, a need for emergent CABG surgery, use of mechanical support and cardiac transplantation, and a higher rate of maternal and fetal mortality in patients with SCAD in peripartum period. Percutaneous coronary interventions (PCI) are associated with a low success rate and high incidence of complications, including iatrogenic dissections and propagation of existing dissections requiring emergency CABG surgery, in the same group of patients (18).

### 4.4 Therapeutic strategies

P-SCAD is potentially the most devastating variant of SCAD. Currently, the scientific community works with limited information about P-SCAD, and a major dilemma is the optimal treatment. While some of the authors suggest that conservative treatment is by far superior to percutaneous intervention (42), there are some scenarios where invasive treatment may be a better option for acute management of P-SCAD (2, 43). The rarity of this entity and the lack of randomized studies, and the complications of invasive treatment make it challenging to choose between conservative management, PCI or CABG (44). The optimal management of SCAD is still unknown. All recommendations are provided by experts' opinions on treating individual cases of SCAD. Conservative management was usually carried out in hemodynamically stable patients without ongoing ischemia or complex angiographic findings- involving the left main coronary artery (45).

Conservative treatment was used in 41.1% (95% CI: 23.2–59.1) of cases. The meta-analysis result indicated that the NP-SCAD patients significantly more frequently received conservative treatment than P-SCAD group (OR = 0.61; 95% CI: 0.37–0.98; *I*^2 ^= 0%) (Figure 5). In the NP-SCAD group, 362 (63.5%) out of 570 women with SCAD underwent conservative treatment. This meta-analysis showed that a non-invasive approach to SCAD treatment is favored for hemodynamically stable patients with NP-SCAD, which confirms the results of previous studies. Although heparin is indicated in patients with ACS, it is recommended to discontinue the anticoagulation therapy after angiographic findings of SCAD to minimize bleeding and enable intramural hematoma to organize (46).

According to the contemporary guidelines, in SCAD patients undergoing subsequent PCI, dual antiplatelet therapy (DAPT) is recommended. The duration of dual antiplatelet therapy after PCI is recommended during 12 months if patients are not on high bleeding risk (46). The optimal duration of monotherapy after 12 months in SCAD patients after PCI remains still unknown (46, 47). The use of dual antiplatelet therapy for 12 months, in SCAD patients, was advocated after the publication of studies where in addition to hematoma, an intraluminal thrombus was frequently found on OCT (47). Since the precise mechanisms of the thienopyridine derivatives elimination route is unknown, the use of clopidogrel is not recommended during breastfeeding (48, 49). Also, prescription of thienopyridine derivatives should be done carefully in premenopausal women due to high risk of menorrhagia (2, 50). As well established, use of low-dose acetylsalicylic acid (<150 mg) in the second and third trimesters is safe (48, 51). There are no randomized studies investigating the use of glycoprotein IIb/IIIa inhibitors for SCAD treatment. In only one study it was noted that the use of glycoprotein IIb/IIIa inhibitors is safe in these patients (52). Therefore, the use of DAPT (but not clopidogrel in breastfeeding women) is recommended in P-SACD and NP- SCAD patients after PCI for 12 months. Single antiplatelet therapy (SAPT) or DAPT, and duration of that therapy, in SCAD patients treated conservatively should be individually tailored comparing the ischemic and bleeding risk. In the DISCO register involving women in high percentage (88.9% overall, and 39.5% of them being post-menopausal), investigators compared the prognosis in patients treated with DAPT vs. SAPT during 12 months in conservatively treated patients with SCAD. In those treated with DAPT compared to those treated with SAPT there was a significantly higher incidence of MACE (all-cause death, non-fatal MI, and any unplanned PCI) (53).

Despite their early usage for SCAD treatment, thrombolytic agents are not recommended because of the risk of dissection expansion and worsening of coronary spasm leading to coronary rupture (2, 54).

Beta-blockers significantly reduce the risk of SCAD recurrence, which can be explained by their role in the reduction of arterial wall stress (54). Nitrates, calcium-channel blockers and ranolazine should be prescribed to relieve chest pain (2, 55). Nitrates are also optimal medication for heart failure, concomitant vasospasm, and residual coronary stenosis (54). Optional agents for left ventricular dysfunction are angiotensin-converting enzyme inhibitors, beta-blockers and mineralocorticoid receptor antagonists (MRA) (47). ACE inhibitors should be carefully prescribed because they are contraindicated in pregnancy and the first month of breastfeeding (32). Statins should be prescribed only for preexisting dyslipidemia because the mechanism of atherosclerosis is not usually associated with SCAD. One small study reported higher statin use in patients with SCAD recurrence (14).

Our results have shown that 60% of females included in this study with P-SCAD are initially presented with STEMI, with high rates of LM and LAD involvement. Of the total number of patients, 41.1% were conservatively treated (95% CI: 23.2–59.1), 32.7% underwent PCI intervention (95% CI: 19.9–45.4), and 3.8% were treated with CABG (95% CI: 0.2–5.7).

In P-SCAD patients, more invasive treatments are performed, typically involving PCI and CABG, vs. a purely conservative approach which was found to be less effective for P-SCAD patients (47). According to the meta-analysis results, the CABG surgery was significantly more frequent in P-SCAD compared to NP-SCAD patients (OR = 6.29; 95% CI: 4.08–9.70; *I*^2 ^= 0%). Included studies have not supplied enough data to perform a meta-analysis about PCI interventions. Factors favoring CABG vs. other therapeutic options are hemodynamic instability, failed PCI, complex coronary anatomy, three-vessel disease, LM involvement, deterioration after the initial conservative approach and ongoing ischemia and SCAD extension in the first 48 h (56).

In the study of Havakuk et al., most of the patients were presented during the postpartum period or the third trimester and none during the first trimester (18). They suggested that timing of presentation should be helpful in predicting SCAD in women with pregnancy-associated myocardial infarction. CABG surgery was done immediately after emergent CS in 6 cases and during pregnancy in 4 women. Fetal mortality was reported in 3 of the cases, all of them in women with LM dissection. Two were related to CABG surgery. Maternal mortality occurred in 5 patients. None of the described cases had a history of conventional cardiovascular disease risk factors, although one woman was diagnosed with Ehler–Danlos syndrome. Four cases presented postpartum and 1 antepartum (18).

The choice between revascularization and conservative therapy for SCAD depends on various factors, including the severity and location of the dissection, the presence of ongoing symptoms, and the patient's hemodynamic conditions. There is ongoing debate and limited evidence regarding the optimal approach, as randomized controlled trials specific to SCAD are scarce (2, 21, 42, 57).

Alfonso et al., in their prospective study of 45 patients, suggested as first-choice a “watchful waiting” approach in stable patients, with a possible switch to revascularization in case of ongoing or recurrent ischemia (51). PCI is accompanied by a risk of adverse events, including an extension of the dissection, guidewire passage into the false lumen and major side branch restriction or occlusion by the propagation of hematoma (47).

It is important to recognize that while P-SCAD is concerning, the prognosis and outcomes can vary widely among individuals. Early recognition, prompt medical intervention, and ongoing support and follow-up care can help manage the condition effectively.

In the most recent retrospective cohort study, Felbaum et al. showed that trends in therapeutic options drastically changed over time (58). In this center, the proportion of patients undergoing revascularization with CABG significantly decreased over a period: 23% of patients were revascularized with CABG before 2013, whereas no patients underwent CABG in 2018–2019. Authors concluded that patients undergoing revascularization with PCI or CABG were more likely to be younger and have pregnancy-associated SCAD, dissection of the left main or left anterior descending artery, and multivessel involvement. This supports the premise that spontaneous arterial healing with conservative management after SCAD is linked to good clinical outcomes (55, 58).

### 4.5 Outcomes

#### 4.5.1 Recurrence rate

The recurrence rate of SCAD in pregnant women has been reported to range from 10% to 29% in various studies (59). Most recurrences tend to happen within the first year after the initial SCAD event, with a peak incidence in the first 4 to 6 months (21). The prevalence of recurrent SCAD was evaluated in 3 of 14 studies. Of 149 pregnant women with spontaneous coronary artery dissection, 6 had recurrent SCAD. In 32 women out of 658 in the reproductive period who were not pregnant, recurrent SCAD was reported. Our meta-analysis result indicated that the prevalence of recurrent SCAD was not significantly more frequent in P-SCAD than in NP-SCAD group, as previously reported.

It is important to note that these rates may not be universally applicable, and individual cases may vary. Due to the limited data on mortality and recurrence rates, specifically in pregnant women with SCAD, medical professionals must provide tailored care and closely monitor patients who experience this condition during pregnancy. Recurrent ischemic events because of persistent or new spontaneous coronary artery dissection are common during long-term follow-up (18, 40).

#### 4.5.2 Mortality rate

Notably, SCAD mortality rates in women are generally lower than those observed in men with traditional atherosclerotic coronary artery disease. However, the risk of mortality in SCAD can still be significant, including the severity and extent of the dissection, underlying risk factors or comorbidities, and the timeliness and effectiveness of medical intervention. Several studies have reported mortality rates ranging from 0% to around 10%, with higher rates in specific subgroups (14).

The mortality and recurrence rates of SCAD in pregnant women are areas of ongoing research, and limited specific data is available. However, several studies have provided insights into these aspects of SCAD in pregnant women. In a retrospective study conducted by Tweet et al., which included 12 pregnant women with SCAD, the overall mortality rate was reported to be 8.3% (60). In a larger retrospective study by Saw et al., which included 87 women with SCAD, 6.9% of the cases occurred during pregnancy (1). The mortality rate in the pregnant group was reported to be 5.3% (33).

Of note, mortality rates may vary among different studies due to differences in patient populations and methodologies. In our analysis of the clinical outcomes, including mortality and non-fatal myocardial infarction (MI) and recurrent SCAD, the prevalence of mortality was 3.3% (95% CI: 1.4–5.1), while estimated prevalence of non-fatal MI and recurrent SCAD was 37.7% (95% CI: 1.9–73.4) and 15.2% (95% CI: 9.1–21.3) (Table 1).

Mortality was evaluated in 3 of 14 studies. Of 149 pregnant women with SCAD, death was the outcome in 6 women, while out of 658 women in the generative period who were not pregnant, 32 died. The meta-analysis result indicated that mortality is not significantly higher in P-SCAD compared to NP-SCAD patients as reported by some studies (18) (Figure 7).

### 4.6 Limitations

This study has several limitations and strengths. Firstly, it was limited to publications available in the English language and was focused on observational studies. Secondly, there was substantial variation in sample sizes across the included studies. Thirdly, high heterogeneity and a limited number of studies prevented a full meta-regression and subgroup meta-analysis; therefore, all findings must be interpreted cautiously. In the proportional meta-analysis, we used random effect due to the heterogeneity of included studies. Unfortunately, sensitivity analysis could not be performed due to the limited number of studies.

## 5 Conclusion

There is great heterogeneity in the methodology of examining the risk for the occurrence of SCAD as well as the decisions for the therapeutic approach in females in the generative period.

Female patients with P-SCAD have more frequently STEMI with involved left main and LAD compared to NP-SCAD patients. Women with NP-SCAD are treated conservatively in higer percentage than P-SCAD patients. Interstingly, P-SCAD compared to NP-SCAD patients do not have significantly higher mortality rates or recurrent coronary dissection.

Developing specialized SCAD registries and research efforts has also contributed to a better understanding of the condition and its outcomes.

## Appendix 1

Search history on Medline 04/04/2023

**Table:**

Search number	Query	Sort by	Filters	Search details	Results	Time
5	((Spontaneous coronary artery dissection) OR (non-atherosclerotic coronary artery dissection)) AND (female)			(((“spontaneous"[All Fields] OR “spontaneously"[All Fields]) AND (“coronary vessels"[MeSH Terms] OR (“coronary"[All Fields] AND “vessels"[All Fields]) OR “coronary vessels"[All Fields] OR (“coronary"[All Fields] AND “artery"[All Fields]) OR “coronary artery"[All Fields]) AND (“dissect"[All Fields] OR “dissected"[All Fields] OR “dissecting"[All Fields] OR “dissection"[MeSH Terms] OR “dissection"[All Fields] OR “dissections"[All Fields] OR “dissects"[All Fields])) OR (“non-atherosclerotic"[All Fields] AND (“coronary vessels"[MeSH Terms] OR (“coronary"[All Fields] AND “vessels"[All Fields]) OR “coronary vessels"[All Fields] OR (“coronary"[All Fields] AND “artery"[All Fields]) OR “coronary artery"[All Fields]) AND (“dissect"[All Fields] OR “dissected"[All Fields] OR “dissecting"[All Fields] OR “dissection"[MeSH Terms] OR “dissection"[All Fields] OR “dissections"[All Fields] OR “dissects"[All Fields]))) AND (“femal"[All Fields] OR “female"[MeSH Terms] OR “female"[All Fields] OR “females"[All Fields] OR “female s"[All Fields] OR “femals"[All Fields])	1,198	03:24:32
4	Non atherosclerotic coronary artery dissection			"non"[All Fields] AND (“atherosclerotic"[All Fields] OR “atherosclerotically"[All Fields] OR “atherosclerotics"[All Fields]) AND (“coronary vessels"[MeSH Terms] OR (“coronary"[All Fields] AND “vessels"[All Fields]) OR “coronary vessels"[All Fields] OR (“coronary"[All Fields] AND “artery"[All Fields]) OR “coronary artery"[All Fields]) AND (“dissect"[All Fields] OR “dissected"[All Fields] OR “dissecting"[All Fields] OR “dissection"[MeSH Terms] OR “dissection"[All Fields] OR “dissections"[All Fields] OR “dissects"[All Fields])	152	03:07:05
3	(Spontaneous coronary artery dissection) AND (female)			(“spontaneous"[All Fields] OR “spontaneously"[All Fields]) AND (“coronary vessels"[MeSH Terms] OR (“coronary"[All Fields] AND “vessels"[All Fields]) OR “coronary vessels"[All Fields] OR (“coronary"[All Fields] AND “artery"[All Fields]) OR “coronary artery"[All Fields]) AND (“dissect"[All Fields] OR “dissected"[All Fields] OR “dissecting"[All Fields] OR “dissection"[MeSH Terms] OR “dissection"[All Fields] OR “dissections"[All Fields] OR “dissects"[All Fields]) AND (“femal"[All Fields] OR “female"[MeSH Terms] OR “female"[All Fields] OR “females"[All Fields] OR “female s"[All Fields] OR “femals"[All Fields])	1,191	03:06:00
2	(Spontaneous coronary artery dissection) AND (pregnancy)			(“spontaneous"[All Fields] OR “spontaneously"[All Fields]) AND (“coronary vessels"[MeSH Terms] OR (“coronary"[All Fields] AND “vessels"[All Fields]) OR “coronary vessels"[All Fields] OR (“coronary"[All Fields] AND “artery"[All Fields]) OR “coronary artery"[All Fields]) AND (“dissect"[All Fields] OR “dissected"[All Fields] OR “dissecting"[All Fields] OR “dissection"[MeSH Terms] OR “dissection"[All Fields] OR “dissections"[All Fields] OR “dissects"[All Fields]) AND (“pregnancy"[MeSH Terms] OR “pregnancy"[All Fields] OR “pregnancies"[All Fields] OR “pregnancy s"[All Fields])	298	03:05:27
1	Spontaneous coronary artery dissection			(“spontaneous"[All Fields] OR “spontaneously"[All Fields]) AND (“coronary vessels"[MeSH Terms] OR (“coronary"[All Fields] AND “vessels"[All Fields]) OR “coronary vessels"[All Fields] OR (“coronary"[All Fields] AND “artery"[All Fields]) OR “coronary artery"[All Fields]) AND (“dissect"[All Fields] OR “dissected"[All Fields] OR “dissecting"[All Fields] OR “dissection"[MeSH Terms] OR “dissection"[All Fields] OR “dissections"[All Fields] OR “dissects"[All Fields])	2,046	03:04:43

Search history on Medline 07/06/2023.

**Table:**

Search number	Query	Sort by	Filters	Search details	Results	Time
9	(((Spontaneous coronary artery dissection) AND ((pregnancy) OR (postpartum) OR (peripartum))) NOT ((case report) OR (review))			((“spontaneous"[All Fields] OR “spontaneously"[All Fields]) AND (“coronary vessels"[MeSH Terms] OR (“coronary"[All Fields] AND “vessels"[All Fields]) OR “coronary vessels"[All Fields] OR (“coronary"[All Fields] AND “artery"[All Fields]) OR “coronary artery"[All Fields]) AND (“dissect"[All Fields] OR “dissected"[All Fields] OR “dissecting"[All Fields] OR “dissection"[MeSH Terms] OR “dissection"[All Fields] OR “dissections"[All Fields] OR “dissects"[All Fields]) AND (“pregnancy"[MeSH Terms] OR “pregnancy"[All Fields] OR “pregnancies"[All Fields] OR “pregnancy s"[All Fields] OR (“postpartum period"[MeSH Terms] OR (“postpartum"[All Fields] AND “period"[All Fields]) OR “postpartum period"[All Fields] OR “postpartum"[All Fields]) OR (“peripartum period"[MeSH Terms] OR (“peripartum"[All Fields] AND “period"[All Fields]) OR “peripartum period"[All Fields] OR “peripartum"[All Fields]))) NOT (“case reports"[Publication Type] OR “case report"[All Fields] OR (“review"[Publication Type] OR “review literature as topic"[MeSH Terms] OR “review"[All Fields]))	97	13:24:27
8	(((Spontaneous coronary artery dissection) AND ((pregnancy) OR (postpartum) OR (peripartum))) NOT (case report)			((“spontaneous"[All Fields] OR “spontaneously"[All Fields]) AND (“coronary vessels"[MeSH Terms] OR (“coronary"[All Fields] AND “vessels"[All Fields]) OR “coronary vessels"[All Fields] OR (“coronary"[All Fields] AND “artery"[All Fields]) OR “coronary artery"[All Fields]) AND (“dissect"[All Fields] OR “dissected"[All Fields] OR “dissecting"[All Fields] OR “dissection"[MeSH Terms] OR “dissection"[All Fields] OR “dissections"[All Fields] OR “dissects"[All Fields]) AND (“pregnancy"[MeSH Terms] OR “pregnancy"[All Fields] OR “pregnancies"[All Fields] OR “pregnancy s"[All Fields] OR (“postpartum period"[MeSH Terms] OR (“postpartum"[All Fields] AND “period"[All Fields]) OR “postpartum period"[All Fields] OR “postpartum"[All Fields]) OR (“peripartum period"[MeSH Terms] OR (“peripartum"[All Fields] AND “period"[All Fields]) OR “peripartum period"[All Fields] OR “peripartum"[All Fields]))) NOT (“case reports"[Publication Type] OR “case report"[All Fields])	172	13:23:36
7	(((Spontaneous coronary artery dissection) AND ((pregnancy) OR (postpartum))) NOT (case report)			((“spontaneous"[All Fields] OR “spontaneously"[All Fields]) AND (“coronary vessels"[MeSH Terms] OR (“coronary"[All Fields] AND “vessels"[All Fields]) OR “coronary vessels"[All Fields] OR (“coronary"[All Fields] AND “artery"[All Fields]) OR “coronary artery"[All Fields]) AND (“dissect"[All Fields] OR “dissected"[All Fields] OR “dissecting"[All Fields] OR “dissection"[MeSH Terms] OR “dissection"[All Fields] OR “dissections"[All Fields] OR “dissects"[All Fields]) AND (“pregnancy"[MeSH Terms] OR “pregnancy"[All Fields] OR “pregnancies"[All Fields] OR “pregnancy s"[All Fields] OR (“postpartum period"[MeSH Terms] OR (“postpartum"[All Fields] AND “period"[All Fields]) OR “postpartum period"[All Fields] OR “postpartum"[All Fields]))) NOT (“case reports"[Publication Type] OR “case report"[All Fields])	148	13:18:40
6	((Spontaneous coronary artery dissection) AND ((pregnancy) OR (postpartum))			(“spontaneous"[All Fields] OR “spontaneously"[All Fields]) AND (“coronary vessels"[MeSH Terms] OR (“coronary"[All Fields] AND “vessels"[All Fields]) OR “coronary vessels"[All Fields] OR (“coronary"[All Fields] AND “artery"[All Fields]) OR “coronary artery"[All Fields]) AND (“dissect"[All Fields] OR “dissected"[All Fields] OR “dissecting"[All Fields] OR “dissection"[MeSH Terms] OR “dissection"[All Fields] OR “dissections"[All Fields] OR “dissects"[All Fields]) AND (“pregnancy"[MeSH Terms] OR “pregnancy"[All Fields] OR “pregnancies"[All Fields] OR “pregnancy s"[All Fields] OR (“postpartum period"[MeSH Terms] OR (“postpartum"[All Fields] AND “period"[All Fields]) OR “postpartum period"[All Fields] OR “postpartum"[All Fields]))	401	13:17:43
5	((Spontaneous coronary artery dissection) AND (pregnancy)) OR (postpartum)			((“spontaneous"[All Fields] OR “spontaneously"[All Fields]) AND (“coronary vessels"[MeSH Terms] OR (“coronary"[All Fields] AND “vessels"[All Fields]) OR “coronary vessels"[All Fields] OR (“coronary"[All Fields] AND “artery"[All Fields]) OR “coronary artery"[All Fields]) AND (“dissect"[All Fields] OR “dissected"[All Fields] OR “dissecting"[All Fields] OR “dissection"[MeSH Terms] OR “dissection"[All Fields] OR “dissections"[All Fields] OR “dissects"[All Fields]) AND (“pregnancy"[MeSH Terms] OR “pregnancy"[All Fields] OR “pregnancies"[All Fields] OR “pregnancy s"[All Fields])) OR (“postpartum period"[MeSH Terms] OR (“postpartum"[All Fields] AND “period"[All Fields]) OR “postpartum period"[All Fields] OR “postpartum"[All Fields])	132,274	13:17:13
4	(Spontaneous coronary artery dissection) NOT (postmenopausal period)			((“spontaneous"[All Fields] OR “spontaneously"[All Fields]) AND (“coronary vessels"[MeSH Terms] OR (“coronary"[All Fields] AND “vessels"[All Fields]) OR “coronary vessels"[All Fields] OR (“coronary"[All Fields] AND “artery"[All Fields]) OR “coronary artery"[All Fields]) AND (“dissect"[All Fields] OR “dissected"[All Fields] OR “dissecting"[All Fields] OR “dissection"[MeSH Terms] OR “dissection"[All Fields] OR “dissections"[All Fields] OR “dissects"[All Fields])) NOT (“postmenopause"[MeSH Terms] OR “postmenopause"[All Fields] OR (“postmenopausal"[All Fields] AND “period"[All Fields]) OR “postmenopausal period"[All Fields])	2,065	13:16:19
3	Spontaneous coronary artery dissection AND postpartum			(“spontaneous"[All Fields] OR “spontaneously"[All Fields]) AND (“coronary vessels"[MeSH Terms] OR (“coronary"[All Fields] AND “vessels"[All Fields]) OR “coronary vessels"[All Fields] OR (“coronary"[All Fields] AND “artery"[All Fields]) OR “coronary artery"[All Fields]) AND (“dissect"[All Fields] OR “dissected"[All Fields] OR “dissecting"[All Fields] OR “dissection"[MeSH Terms] OR “dissection"[All Fields] OR “dissections"[All Fields] OR “dissects"[All Fields]) AND (“postpartum period"[MeSH Terms] OR (“postpartum"[All Fields] AND “period"[All Fields]) OR “postpartum period"[All Fields] OR “postpartum"[All Fields])	222	13:14:09
2	(Spontaneous coronary artery dissection) AND (pregnancy)			(“spontaneous"[All Fields] OR “spontaneously"[All Fields]) AND (“coronary vessels"[MeSH Terms] OR (“coronary"[All Fields] AND “vessels"[All Fields]) OR “coronary vessels"[All Fields] OR (“coronary"[All Fields] AND “artery"[All Fields]) OR “coronary artery"[All Fields]) AND (“dissect"[All Fields] OR “dissected"[All Fields] OR “dissecting"[All Fields] OR “dissection"[MeSH Terms] OR “dissection"[All Fields] OR “dissections"[All Fields] OR “dissects"[All Fields]) AND (“pregnancy"[MeSH Terms] OR “pregnancy"[All Fields] OR “pregnancies"[All Fields] OR “pregnancy s"[All Fields])	302	13:13:16
1	Spontaneous coronary artery dissection			(“spontaneous"[All Fields] OR “spontaneously"[All Fields]) AND (“coronary vessels"[MeSH Terms] OR (“coronary"[All Fields] AND “vessels"[All Fields]) OR “coronary vessels"[All Fields] OR (“coronary"[All Fields] AND “artery"[All Fields]) OR “coronary artery"[All Fields]) AND (“dissect"[All Fields] OR “dissected"[All Fields] OR “dissecting"[All Fields] OR “dissection"[MeSH Terms] OR “dissection"[All Fields] OR “dissections"[All Fields] OR “dissects"[All Fields])	2,074	13:12:38
